# An Increase in Peripheral Temperature following Cocaine Administration Is Mediated through Activation of Dopamine D2 Receptor in Rats

**DOI:** 10.3390/life12020143

**Published:** 2022-01-19

**Authors:** Suchan Chang, Yeonhee Ryu, Se Kyun Bang, Han Byeol Jang, DanBi Ahn, Hyung Kyu Kim, Hubert Lee, Sang Chan Kim, Bong Hyo Lee, Hee Young Kim

**Affiliations:** 1 Department of Physiology, College of Korean Medicine, Daegu Haany University, Daegu 42158, Korea; c64111915@gmail.com (S.C.); star961018@naver.com (H.B.J.); an951121@dhu.ac.kr (D.A.); badawanabi@gmail.com (H.K.K.); dlqhdgy@dhu.ac.kr (B.H.L.); 2 Korean Medicine Fundamental Research Division, Korea Institute of Oriental Medicine, Daejeon 34054, Korea; yhryu@kiom.re.kr (Y.R.); sichosi@kiom.re.kr (S.K.B.); 3 Department of Pharmacology and Toxicology, University of Texas Medical Branch, 301 University Boulevard, Galveston, TX 77555, USA; hulee@utmb.edu; 4 Medical Research Center, College of Korean Medicine, Daegu Haany University, Gyeongsan 38610, Korea; sckim@dhu.ac.kr

**Keywords:** cocaine, increased temperature, peripheral body temperature, dopamine D2 receptor, bromocriptine

## Abstract

Drug addiction has become a worldwide problem, affecting millions of people across the globe. While the majority of mechanistic studies on drug addiction have been focused on the central nervous system, including the mesolimbic dopamine system, the peripheral actions of drugs of abuse remain largely unknown. Our preliminary study found that the systemic injection of cocaine increased peripheral skin temperature. This led us to our present study, which investigated the mechanisms underlying the increase in peripheral temperature following cocaine injection. Male Sprague Dawley rats were anesthetized with pentobarbital sodium, and peripheral skin temperature measurements were taken using a thermocouple needle microprobe and an infrared thermal camera. Cocaine injection caused an acute rise in peripheral body temperature, but not core body temperature, about 10 min after injection, and the temperature increases were occluded by systemic injection of dopamine D2 receptor antagonist L741,626, but not D1 receptor antagonist SCH23390. In addition, systemic administration of bromocriptine, a dopamine D2 receptor agonist, significantly increased peripheral temperature. Infrared thermal imaging showed that the thermal increases following cocaine injection were predominantly in the distal areas of the forelimbs and hindlimbs, relative to core body temperature. Treatment with cocaine or bromocriptine decreased the size of skin blood vessels without affecting the expression of dopamine D2 receptors. These results suggest that increased peripheral temperature in skin following cocaine injection is associated with the activation of the dopamine D2 receptor.

## 1. Introduction

Cocaine addiction is a chronic, relapsing neuropsychiatric disorder characterized by compulsive risk-taking and reward-seeking behaviors. In 2019, approximately 20 million people worldwide had used cocaine within the past year [1]. The central mechanisms underlying drug addiction have been investigated in various ways [2]. Cocaine is rooted as a highly addictive psychostimulant with a wide range of effects. Specifically, it targets and blocks the dopamine transporter (DAT) in the mesolimbic reward system, causing a rapid increase in extracellular dopamine levels and the subsequent activation of dopamine receptors, the post-synaptic D1 receptors and the pre- and post-synaptic D2 receptors, which produces reinforcing effects that lead to cocaine abuse [3,4]. In addition to these reinforcing properties, cocaine also produces systemic effects in humans, such as increased heart rate and blood pressure and respiratory depression [5,6]. Previous studies have shown that cocaine contributes to cardiovascular diseases in humans [7]. It stimulates the sympathetic nervous system by inhibiting catecholamine reuptake at the sympathetic nerve terminals [8,9], and thus increases heart rate and blood pressure [10,11]. 

While most studies on drug addiction have focused on the central nervous system, including the mesolimbic dopamine reward system, or systemic effects, the peripheral actions of drugs of abuse remain largely unknown. Because the peripheral nervous system activates the reward system, the peripheral system is believed to be the initial component of cocaine’s systemic rewarding effects in relapsed users [12]. Cocaine is reported to be associated with a number of skin alterations, such as urticarial vasculitis, retiform purpura, scleroderma, and Raynaud’s phenomenon, likely secondary to vasoconstriction and ischemia in humans [13]. Dental decay and vasoconstriction of the oral mucosal vasculature have been observed in methamphetamine users [13]. Heroin or alcohol abusers have reported pruritic urticarial rashes across the body [14]. 

In the present study, we hypothesized that cocaine administration would cause physiological changes to the peripheral regions, resulting in changes in skin temperature via the activation of the dopamine system. To explore this, we investigated whether (1) acute administration of cocaine changes peripheral skin or core body temperature in rats, (2) the increased peripheral skin temperature following cocaine injection is prevented by pretreatment with certain dopamine receptor antagonists, (3) dopamine receptor agonists can elevate peripheral body temperature, and (4) cocaine or a dopamine D2 agonist affect the size of skin blood vessels, or the expression of dopamine D2 receptors.

## 2. Materials and Methods

### 2.1. Animals

Since female rats typically exhibit estrous cycles, which affects core body temperature [15], they were excluded from the study. Male Sprague Dawley rats (weight 270–320 g, 7–8 weeks old, Daehan Animal, Seoul, Korea) were used in this study. Each cohort consisted of 5–6 rats. Home cages were kept under a 12 h light–dark cycle at a temperature of 22 ± 2 °C and humidity of 40–60%, with free access to food and water. All procedures were carried out in accordance with the National Institutes of Health Guide for the Care and Use of Laboratory Animals, and approved by the Institutional Animal Care and Use Committee at Daegu Haany University (DHU 2021-006). 

### 2.2. Chemicals

The following chemicals were used: cocaine hydrochloride (20 mg/kg, dissolved in saline, intraperitoneal (i.p.), MacFarlan Smith Ltd., Edinburgh, UK); SCH23390 (10 µg/kg, dissolved in saline, i.p., a dopamine D1 receptor antagonist, Sigma-Aldrich, St. Louis, MO, USA); L741,626 (3 mg/kg, dissolved in saline, i.p., a dopamine D2 receptor antagonist, Sigma-Aldrich, St. Louis, MO, USA); bromocriptine (5 mg/kg, dissolved in 5% dimethyl sulfoxide (DMSO), i.p., a dopamine D2 receptor agonist, Abcam, Cambridge, UK). The doses of SCH23390, L741,626, and bromocriptine were based on those from previous studies [16,17,18]. 

### 2.3. Measurement of Peripheral Body and Core Body Temperatures

Peripheral body temperature was measured over either the left or right wrist (one spot per animal) using a thermocouple needle microprobe (NJ-07013, Sarasota, FL, USA) coupled with an analog–digital interface converter (Physitemp BAT-12, American Laboratory Trading, San Diego, CA, USA). Core body temperature was monitored using a rectal thermocouple probe. The signals from the thermocouple probe were digitized through a PowerLab 4/30 acquisition system (ADInstruments, Colorado Springs, CO, USA). While the rats were anesthetized with pentobarbital (50 mg/kg, i.p.) and maintained at a temperature of 37 °C using a thermostatically controlled heating pad (TC-1000 Temperature controller, CWE Inc., Ardmore, PA, USA), the needle microprobe thermocouple was attached to the skin over the wrist. After recording the basal temperature for 10 min, the animals were given an intraperitoneal injection of cocaine (20 mg/kg) and monitored for up to 30 min after injection. During the experiment, infrared thermal images were obtained using a thermal camera (TE-M1-PLUS, i3system Inc., Daejeon, Korea). SCH23390 (10 µg/kg) or L741,626 (3 mg/kg) were intraperitoneally injected 10 min before cocaine administration. 

### 2.4. Immunohistochemistry for Dopamine D2 Receptor

Separate cohorts of animals were euthanized 30 min after saline, cocaine, or bromocriptine injection. Skin samples were taken from the wrist and post-fixed with 4% paraformaldehyde. The skin samples (one sample from left or right wrist per rat) were paraffin-embedded, sectioned (5 μm), and incubated with primary antibodies (1:1000, anti-dopamine D2 receptor rabbit polyclonal, Cat#: AB5084P, EMD Millipore Corp, Burlington, MA, USA; for specificity, see [19,20]), followed by incubation with a secondary antibody (1:500, Alexa Fluor 488-conjugated donkey anti-rabbit IgG antibody, Invitrogen, Waltham, MA, USA). The sections were mounted onto gelatine-coated slides, air-dried, and coverslipped with a mounting medium (Vector, Cat# H-1000). Immunoreactivity of dopamine D2 receptors was visualized at a 480 nm wavelength, and photographs were taken of three sections from each skin sample with an epifluorescence microscope (magnification 100×, Olympus BX51, Tokyo, Japan). The mean intensity of green fluorescence in the dermis of the wrist was measured using ImageJ software (National Institute of Mental Health, Bethesda, MD, USA). 

### 2.5. Histological Assessment of Skin Blood Vessels

All rats were euthanized for histological examination 30 min after saline, cocaine, or bromocriptine injection. Animals were perfused with phosphate-buffered saline (PBS) and then with 4% paraformaldehyde. Skin samples (one sample from left or right wrist per rat) were taken from the wrist, paraffin-embedded, sectioned into 5 μm-thick segments and stained with hematoxylin and eosin (H&E). The images were captured using a digital camera connected to a microscope (magnification 100×, Olympus BX51, Tokyo, Japan). The dermal blood vessels in the images were identified by their internal cavities, endothelial lining, and sometimes the containment of red blood cells. The cross-sectional areas (µm^2^) of the internal cavities in the dermal blood vessels were measured using a digital image program (Olympus, cellSens Dimension 1.18, Tokyo, Japan). 

### 2.6. Statistical Analysis

Statistical analysis was carried out using SigmaStat 3.5 software (Systat Software, Inc., Chicago, IL, USA). Images were analyzed by an independent investigator blinded to treatment assignment. All data are presented as the mean ± SEM (standard error of the mean) and analyzed by a one-way or two-way repeated measures analysis of variance (ANOVA) with Tukey post hoc tests. Statistical significance was considered at *p* < 0.05. By using the SigmaStat 3.5 software, the sample size of 5–6 animals per group was estimated to have the statistical power of ≥0.8 for within–between interactions, with an effect size of 0.33 at alpha = 0.05 (correlation among repeated measures = 0.5 and nonsphericity correction epsilon).

## 3. Results

### 3.1. An Increase in Peripheral Body Temperature following Cocaine Injection

**Figure 1:**
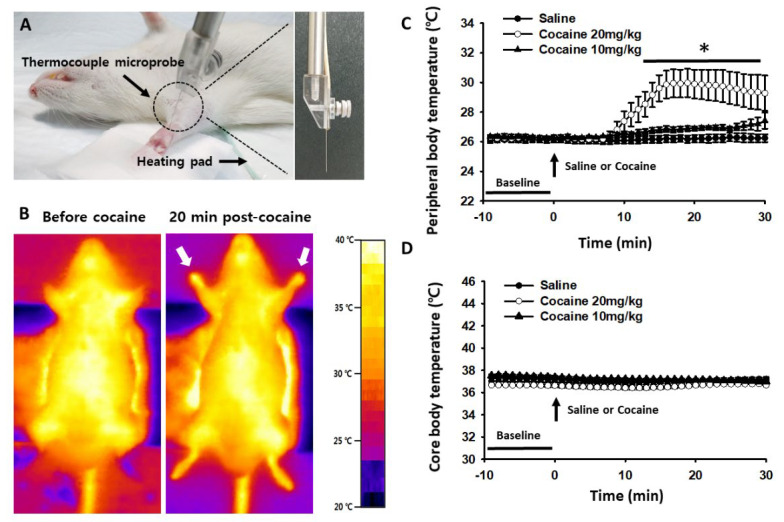
An increase in peripheral body temperature following systemic injection of cocaine. (**A**) Representative images for measurement of peripheral body temperature (left) and needle thermocouple microprobe (right); (**B**) Representative thermal infrared images of saline- and cocaine (20 mg/kg)-treated rats; (**C**) Effect of cocaine injection on peripheral skin (wrist) temperature. Systemic injection of cocaine at the dose of 20 mg/kg (cocaine 20 mg/kg, *n* = 5), but not 10 mg/kg (cocaine 10 mg/kg, *n* = 5), significantly increased peripheral body temperature compared to saline-injected group (saline, *n* = 6). * *p* < 0.05 vs. Saline; (**D**) Effect of cocaine injection on core body temperature.

To test whether cocaine changes peripheral or core body temperature, temperatures of the wrist and rectum were monitored following acute administration of cocaine or saline, respectively (Figure 1A). Peripheral body temperature over the wrist significantly and rapidly increased 10 min after cocaine injection at the higher dose of 20 mg/kg, but temperature changes were not observed at the lower dose of 10 mg/kg when compared to the values before cocaine injection or in the saline-injected group. The 20 mg/kg dose effects lasted for at least 30 min after injection (two-way repeated ANOVA; group F_(1, 4)_ = 6.526, *p* = 0.063; time F_(29, 116)_ = 10.440, *p* < 0.001; interaction F_(29, 116)_ = 7.772, *p* < 0.001; Figure 1C). Conversely, core body temperature remained unaffected following administration of either cocaine or saline (Figure 1D). 

To further confirm the elevation of peripheral skin temperature, whole body thermal images were taken using a high-resolution infrared thermal camera, before and 20 min after injection, in the saline- and cocaine-treated groups. From the thermal images, the elevation of temperature following cocaine injection was found predominantly in the peripheral skin of the forelimbs and hindlimbs, while the central body and trunk skin temperatures showed little change (Figure 1B). Taken together, the results show that cocaine caused a marked increase in peripheral body temperature. 

### 3.2. Involvement of Dopamine Receptors in Cocaine-Induced Elevation of Peripheral Skin Temperature

**Figure 2:**
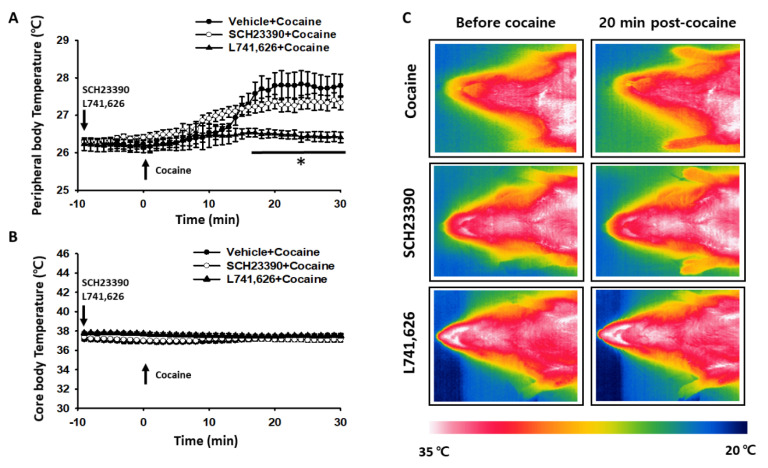
Effect of systemically injected D1 or D2 receptor antagonist on cocaine-induced peripheral body temperature. (**A**) Effect of pretreatment with dopamine D1 or D2 receptor antagonist on cocaine (20 mg/kg)-induced peripheral body temperature. SCH23390 (D1 receptor antagonist, *n* = 6), L741,626 (D2 receptor antagonist, *n* = 6) or saline (*n* = 6) was intraperitoneally injected 10 min before cocaine administration. * *p* < 0.05 vs. Vehicle + Cocaine; (**B**) Effect of pretreatment with dopamine D1 or D2 receptor antagonist on core body temperature in cocaine-treated rats. There are no differences in the core body temperature changes among the groups; (**C**) Representative thermal infrared images.

To explore the involvement of the dopamine system in the cocaine-induced increase in peripheral body temperature, dopamine receptor antagonists were injected prior to cocaine injection. Under sodium pentobarbital anesthesia, rats were given either vehicle, SCH23390 (D1 receptor antagonist) or L741,626 (D2 receptor antagonist) 10 min before cocaine injection. Peripheral skin temperature significantly increased following cocaine injection (saline + cocaine) compared to the values before cocaine injection. This effect was almost completely blocked by intraperitoneal injection of L741,626, but not SCH23390 (two-way repeated ANOVA; group F_(2, 8)_ = 6.038, *p* = 0.025; time F_(29, 116)_ = 18.536, *p* < 0.001; interaction F_(58, 232)_ = 5.921, *p* < 0.001; Figure 2A). Neither SCH23390 nor L741,626 affected core body temperature (Figure 2B). Inhibition by LH741,626 of cocaine-induced peripheral body temperature was further confirmed by the infrared thermal imaging (Figure 2C). Overall, the results show that the cocaine-induced increase in peripheral body temperature was mediated by D2 receptors. In addition, our results show that the antagonistic manipulation of D1 receptors did not affect peripheral body temperature at the tested concentration.

### 3.3. Increase in Peripheral Body Temperature by Dopamine D2 Receptor Agonist

**Figure 3:**
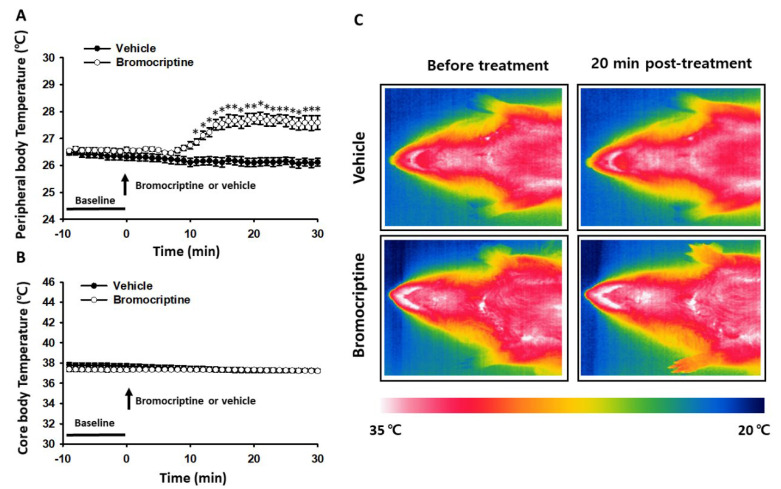
An increase in peripheral body temperature following systemic injection of dopamine D2 receptor agonist bromocriptine. (**A**) Effect of bromocriptine injection on peripheral body temperature. Systemic injection of bromocriptine (*n* = 6) significantly increased peripheral body temperature (measured in wrist skin) compared to vehicle-injected group (*n* = 6). * *p* < 0.05 vs. Vehicle (5% DMSO); (**B**) Effect of bromocriptine on core body temperature; (**C**) Representative thermal infrared images of bromocriptine- and vehicle-treated rats.

To further confirm the involvement of dopamine D2 receptors in cocaine-induced increases in peripheral body temperature, we tested whether activation of dopamine D2 receptors itself increases the peripheral body temperature in naïve rats. After recording basal temperature, rats were given an intraperitoneal injection of bromocriptine (a dopamine D2 receptor agonist) and monitored for up to 30 min after injection. Peripheral body temperature, but not core body temperature, significantly increased following bromocriptine injection, while both core body temperature and peripheral body temperature following vehicle injection remained unchanged (two-way repeated ANOVA; group F_(1, 5)_ = 27.572, *p* = 0.003; time F_(29, 145)_ = 10.531, *p* < 0.001; interaction F_(29, 145)_ = 21.920, *p* < 0.001; Figure 3A,B). Thermal infrared imaging further confirmed elevated temperatures in the skin over the wrist 20 min after bromocriptine injection (Figure 3C). 

### 3.4. Histological Measurement and Dopamine D2 Receptor Expression on Peripheral Skin Area

**Figure 4:**
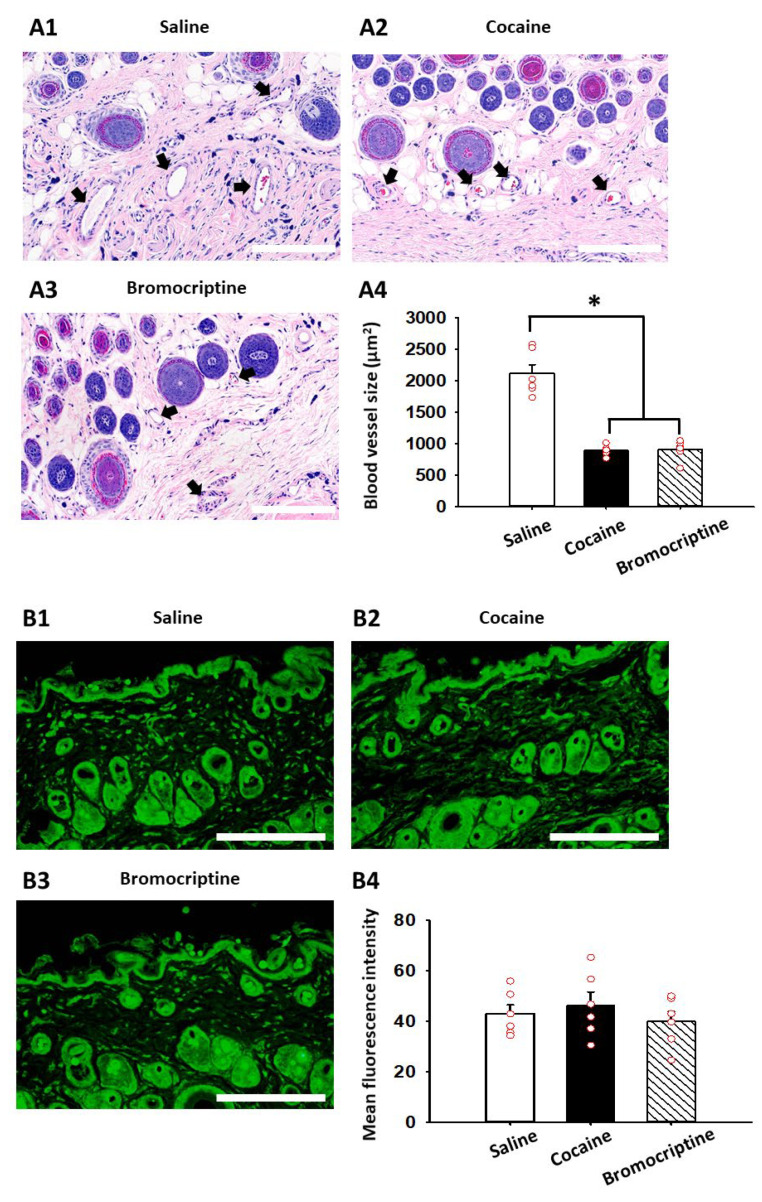
Histological and immunohistochemical examination of peripheral skin in cocaine- and dopamine D2 receptor agonist bromocriptine-treated rats. (**A**) Histological examination of blood vessel sizes in the peripheral skin area in saline-, cocaine-, and bromocriptine-treated rats (**A1**–**A3**) Systemic injection of cocaine (*n* = 6) and bromocriptine (*n* = 6) significantly decreased blood vessel sizes compared to saline (*n* = 6); (**A4**) Scale bar = 200 μm, * *p* < 0.05 vs. Saline; (**B**) Dopamine D2 receptor expression in peripheral skin area (wrist). There were no differences in the fluorescence intensity of D2 receptor expression among the groups; (**B1**–**B4**) *n* = 6/group, scale bar = 200 μm, a.u. = arbitrary unit.

We further explored whether cocaine or bromocriptine causes the morphological changes in skin blood vessels in the peripheral skin. From histological examination, we found that the blood vessel sizes in the peripheral skin area significantly decreased in cocaine- and bromocriptine-treated groups compared to those of the saline-treated rats (one-way ANOVA; F_(2, 15)_ = 56.348, *p* < 0.001; Figure 4(A1–A4)). Since it was previously reported that cocaine administration alters the expression of dopamine D2 receptors in the brain reward systems [21,22], we also analyzed the expression of dopamine D2 receptors in the peripheral skins of cocaine- and bromocriptine-treated rats. In a quantitative immunohistochemical analysis, no changes were found in the dopamine D2 receptor expression in the peripheral skin among the saline-, cocaine-, and bromocriptine-treated groups (Figure 4(B1–B4)). 

## 4. Discussion

The present study is the first to report the phenomenon of peripheral body temperature increase following systemic injection of cocaine. Cocaine injection induced an acute increase in peripheral body temperature, while core body temperature was maintained at 37 °C. The increased peripheral temperature was arrested by dopamine D2 receptor antagonist L741,626, but not D1 receptor antagonist SCH23390. When a dopamine D2 receptor agonist, bromocriptine, was injected, an increase in peripheral body temperature was observed in a similar pattern as that in cocaine-injected rats. The injection of cocaine or bromocriptine reduced the sizes of skin surface blood vessels without affecting the expression of peripheral dopamine D2 receptors. The results suggest the involvement of dopamine D2 receptors in cocaine-induced increases in peripheral body temperature. 

The present study showed a consistent result that systemic administration of cocaine (20 mg/kg) increased peripheral skin temperature in rats. On the other hand, Sullivan et al. reported that low doses (i.e., 0.3–0.8 mg/kg) of cocaine in human subjects decreased both peripheral blood flow and skin temperature, occurring at about 14 min after injection [23], and suggested that the decrease in skin temperature might be due to cocaine’s vasoconstrictive properties. To date, there have been few clinical or experimental studies that show changes in peripheral temperature at the cocaine doses required to produce psychomotor responses or euphoric effects. Based on our present and previous studies [23], a high dose of cocaine may increase peripheral temperature, but a low dose of cocaine may actually reduce peripheral temperature. The cocaine-induced rise in peripheral temperature was inhibited by blocking dopamine D2 receptors and mimicked by the administration of dopamine D2 receptor agonist bromocriptine. It is also known that bromocriptine interacts with other dopamine receptors and with various serotonin and adrenergic receptors [24,25,26]. Although the innate pharmacological properties of bromocriptine cannot exclude the involvement of other receptors, these results may suggest that cocaine induces an increase in peripheral temperature at a sublethal dose (20 mg/kg) in rats, and that the effects are generated through mechanisms including the possible activation of dopamine D2 receptors. 

Previous clinical studies revealed that when subjects received cocaine at a normal body temperature (about 37 °C), it induced various physical reactions, such as increases in blood pressure, heart rate and breathing rate and other peripheral actions [7]. In humans, cocaine can lead to heat dissipation disorders, such as sweating dysfunctions, skin vasodilations, and changes in heat perception [27]. It was also reported that cocaine causes ambient-temperature-dependent body temperature changes in rats [28]. At a 20 °C ambient temperature, cocaine (10–40 mg/kg, i.p.) induced a dose-dependent fall in body temperature, while a dose of cocaine (20 mg/kg, i.p.) at a 35 °C ambient temperature caused a small increase (about 0.6 °C) in core body temperature in conscious rats [28]. Ferguson et al. demonstrated that injection of dopamine (20 ug) into the hypothalamus of rats caused a fall in core temperature in control animals, but a rise in animals raised at, or acclimated to, a higher ambient temperature of 33 °C [insert reference]. Moreover, Lin et al. reported that electrical stimulation of the substantia nigra induced hypothermia, decreased metabolism, and cutaneous vasoconstriction in rats maintained at ambient temperatures below 22 °C, and induced hyperthermia and cutaneous vasoconstriction in rats at an ambient temperature of 30 °C. It is known that peripheral vessel tones and temperature regulation of the hypothalamus are critically influenced by ambient temperature [29,30]. Thus, cocaine-induced increases in peripheral temperature, at ambient temperatures above 30 °C may be due to the combined effects of cocaine, such as increased muscular activity and peripheral vasoconstriction, and/or its direct effect on dopamine-modulated, heat-regulatory centers in the hypothalamus, which remains to be explored. On the other hand, the present study showed that acute administration of cocaine (20 mg/kg, i.p.) did not cause significant changes in the core body temperatures of pentobarbital-anesthetized rats, which were maintained at 37 °C on a heating pad. This is consistent with previous studies that show injection of cocaine (20 mg/kg) does not modify the core body temperature in rats [31]. The data showed that systemic administration of cocaine at a dose of 20 mg/kg elevated peripheral body temperature without affecting core body temperature in anesthetized rats maintained at 37 °C using a heating pad. The animals were under pentobarbital anesthesia and were warmed on a heating pad. It was reported that sodium pentobarbital (50 mg/kg) decreases core body and skin temperatures by reducing the metabolism of brain thermointegrative centers, including the hypothalamus and hippocampus, and these changes are partially compensated by external body warming with a heating pad [32]. However, this may not exclude the possibility that the effect of cocaine on core body temperature is offset by the conditions provided (i.e., pentobarbital or heating pad), and that the rise in peripheral body temperature seen in our setup might arise as a consequence of this.

In the present study, the cocaine-induced increase in peripheral temperature was abolished by pretreatment with a D2 receptor antagonist, but not with a D1 receptor antagonist, and mimicked by activating the dopamine D2 receptor, suggesting the involvement of dopamine D2 receptors in the rise in peripheral body temperature following cocaine injection. Cocaine is known to block dopamine transporters, resulting in subsequent increases in extracellular dopamine levels that activate dopamine D1 and D2 receptors, generating various biological actions, such as cardiovascular abnormalities and reinforcing effects [33,34]. Dopamine receptors are also found in the skin of humans and animals [35]. It is known that dopamine D1 receptors are distributed particularly in the dermis layer of skin, and D2 receptors are prominent in the subcutaneous tissue near the vessels of humans and mice [36,37]. Dopamine plays a role in skin barrier homeostasis by acting on dopamine D2 receptors in the skin, and the application of D2-like receptor agonists, such as bromocriptine, accelerates skin barrier recovery [37]. Stimulation of D2 receptors activates the intracellular phospholipase C signaling pathway [38]. Increased levels of phospholipase C in the skin have been shown to induce vasodilation, edema, and inflammation [39,40]. Thus, we can assume that the dopamine levels released by cocaine stimulate the D2 receptors in subcutaneous tissue, resulting in an increase in peripheral temperature by modulating phospholipase C signaling. Another possible explanation is an interaction between dopamine D2 and serotonin (5-HT) receptors. Dopamine directly activates serotonin receptors, such as 5-HT1A, 5-HT2C, and 5-HT3, in HEK293 cells [41], although the potency and efficacy of dopamine binding vary among the subtypes, and the affinity of the ligand to 5-HT receptors does not correlate with the efficacy of the ligand for activation [42]. Previous studies have demonstrated that dopamine exerts a facilitatory effect on 5-HT neurotransmission, and D2 receptor activation can excite 5-HT cells [43]. In one such study, systemic injection of 5-HT agonists produced hyperthermia, which was inhibited by 5-HT receptor antagonists [44,45,46]. Thus, an interaction between 5-HT and D2 receptors may contribute to a rise in peripheral temperature following cocaine administration, which remains to be explored. 

In conclusion, cocaine injection induces an increase in peripheral body temperature by activating dopamine D2 receptors. 

## Data Availability

The data used to support the findings of this study are included in the manuscript.
